# A Functional Variant at miR-520a Binding Site in PIK3CA Alters Susceptibility to Colorectal Cancer in a Chinese Han Population

**DOI:** 10.1155/2015/373252

**Published:** 2015-03-05

**Authors:** Lifang Ding, Zao Jiang, Qiaoyun Chen, Rong Qin, Yue Fang, Hao Li

**Affiliations:** ^1^Department of Oncology, Danyang People's Hospital, Danyang 213000, China; ^2^Department of Oncology, Affiliated Zhongda Hospital of Southeast University, Nanjing 210009, China; ^3^Department of Central Laboratory, Affiliated People's Hospital of Jiangsu University, Zhenjiang 212002, China; ^4^Department of Oncology, Affiliated People's Hospital of Jiangsu University, Zhenjiang 212002, China; ^5^Department of Clinical Laboratory, Taixing People's Hospital, Taixing 225400, China

## Abstract

An increasing body of evidence has indicated that polymorphisms in the miRNA binding site of target gene can alter the ability of miRNAs to bind their target genes and modulate the risk of cancer. We aimed to investigate the association between a miR-520a binding site polymorphism rs141178472 in the PIK3CA 3′-UTR and the risk of colorectal cancer (CRC) in a Chinese Han population. The polymorphism rs141178472 was analyzed in a case-control study, including 386 CRC patients and 394 age- and sex-matched controls; the relationship between the polymorphism and the risk of colorectal cancer was examined. Individuals carrying the rs141178472 CC genotype or C allele had an increased risk of developing CRC (CC versus TT, OR (95% CI): 1.716 (1.084–2.716), *P* = 0.022; C versus T, OR (95% CI): 1.258 (1.021–1.551), *P* = 0.033). Furthermore, the expression of PIK3CA was detected in the peripheral blood mononucleated cell of CRC patients, suggesting that mRNA levels of PIK3CA might be associated with SNP rs141178472. These findings provide evidence that a miR-520a binding site polymorphism rs141178472 in the PIK3CA 3′-UTR may play a role in the etiology of CRC.

## 1. Introduction

Colorectal cancer is the third most common malignant disease worldwide and is a major cause of morbidity and mortality throughout the world [1]. During the past few decades, a rapid increase in the incidence and mortality of colorectal cancer has been reported in China [2]. Colorectal carcinogenesis is a comprehensive, multifactorial, and multistep process which is caused by the interaction of environmental agents and genetic susceptibility [3, 4]. The mechanism of colorectal carcinogenesis remains still not fully understood. Despite environmental agents found to be major risk factors for colorectal cancer, only a fraction of individuals exposed to the same risk factors develop colorectal cancer during their lifetime, suggesting that other factors were associated with the development of colorectal cancer. In recent years, an increasing body of evidence suggests that genetic polymorphisms modulate the risk of carcinogenesis and that genetic susceptibility plays an important role in the occurrence of human cancers [5, 6].

MicroRNAs (miRNAs) are a class of single-stranded 21–23-nucleotide- (nt) long endogenous noncoding RNAs that negatively regulate target gene expression at the posttranscriptional level [7]. Due to the influence on miRNA and/or their target gene expression, single nucleotide polymorphisms (SNPs) in microRNA (miRNA) genes and the miRNA binding sites at the 3′ untranslated region (UTR) of their target genes play an important role in the cancer susceptibility. PIK3CA gene that encoded the catalytic p110-alpha subunit of PI3K has been described to be commonly mutated in various cancers, including colorectal cancer [8]. A miR-520a binding site polymorphism rs141178472 was found located at the PIK3CA 3′-UTR using bioinformatics analysis. But the association between this polymorphism and colorectal carcinogenesis remains unclear.

Given the role of microRNA and PIK3CA in carcinogenesis, we hypothesized that genetic variations in the PIK3CA 3′-UTR may confer individual susceptibility to colorectal cancer. Here, we conducted a case-control study to investigate the association of a miR-520a binding site polymorphism rs141178472 in the PIK3CA 3′-UTR with the risk of colorectal cancer in a Chinese population.

## 2. Materials and Methods

### 2.1. Study Populations

The study population consisted of 386 cases with CRC (age range, 23–72 years) and 394 controls (age range, 21–73 years). The present study is a hospital-based case-control study. Cases were the patients with pathological confirmed CRC and were consecutively recruited from Danyang People's Hospital and Zhenjiang First People's Hospital, Jiangsu. The control subjects were randomly selected from a pool of healthy individuals who got a routine health checkup. All cases and control subjects were genetically unrelated and control subjects had no individual history of cancer. All study participants signed the written informed consent. Demographic information and environmental exposure history were obtained from the participants using a standardized questionnaire. The present study was approved by the Institutional Review Board of Danyang People's Hospital and the Affiliated People's Hospital of Jiangsu University.

### 2.2. Genotyping

Genomic DNA was extracted with the QIAamp DNA Mini Kit (Qiagen) from isolated peripheral blood lymphocytes. The PIK3CA rs141178472 was genotyped using the allele-specific PCR assay on the S1000 thermal cycler (Bio-Rad). The primers for rs141178472 were shown in Table 1. In each 20 *μ*L reaction, 100 ng genomic DNA was amplified by 1.5U Taq DNA polymerase (Takara) with 0.5 *μ*L of each primer and 2 *μ*L of 2.5 mM dNTPs. The PCR thermal cycling amplification was performed under the following conditions: 95°C for 3 min, 35 cycles of 95°C for 30 sec, 60°C for 30 sec, 72°C for 30 sec, and 72°C for 10 min. After the amplification, electrophoresis was performed at 80 V for 60 min in 0.5X Tris-borate-EDTA buffer on 1% agarose gel stained with ethidium bromide (0.5 *μ*g/*μ*L). After electrophoresis, the amplified products were visualized under UV light.

### 2.3. PIK3CA 3′-UTR Luciferase Reporter Plasmid Construction and Transfection

The 3′-UTR region of PIK3CA containing the putative recognition site rs141178472 was amplified from a DNA sample carrying CC genotype. The primers were 5′-TCATGGTGGCTGGACAACAA-3′ (sense) and 5′-TCCAAAGCTTTACTGGTGTGAGCCACTGTG-3′ (antisense). PCR products were separated in 0.8% agarose gel, extracted, purified, and then subcloned into the pMIR-REPORT (Applied Biosystems) vector. Plasmid containing the rs141178472 T allele was generated using a QuikChange Site-Directed Mutagenesis Kit (Invitrogen). The 293T cells were maintained in Dulbecco's modified Eagle's medium supplemented with 10% calf serum, 50 U/mL penicillin, and 50 *μ*g/mL streptomycin at a 37°C incubator supplemented with 5% CO_2_. Transfections were performed with cells using Lipofectamine 2000 according to manufacturer's instruction (Invitrogen) after 24 h. The PIK3CA 3′-UTR luciferase plasmids (C allele or T allele) and chemically synthesized mature miR-520a were cotransfected into 293T cells, respectively. After 24 h of incubation, cells were collected and processed for luciferase assay with dual-luciferase reporter assay kit (Promega Corporation).

### 2.4. qRT-PCR for Expression of PIK3CA

Total RNA was extracted from peripheral blood mononuclear cells (PBMCs) of 60 patients using Trizol reagent (Invitrogen, USA) according to the manufacturer's instructions. Quantitative real-time polymerase chain reaction (qRT-PCR) for PIK3CA was performed on Bio-Rad CFX96 Thermal Cycler using SYBR Green Q-PCR Master Mix (Takara, Dalian, China). The primers used in qRT-PCR were as follows: 5′-GGAGCCTGGAAGAGCCC-3′ (F); 5′-CGTGGAGGCATTGTTCTGAT-3′ (R). The qRT-PCR was performed as described previously [9].

### 2.5. Statistical Analysis

All statistical analyses were performed using SPSS version 12.0 (SPSS, Chicago, IL, USA) with a two-sided test. The studied polymorphism rs141178472 was tested for Hardy-Weinberg equilibrium (HWE) among the controls. The frequencies of alleles and genotypes between patients and controls were compared using *χ* ^2^ test or Fisher's exact test. Odds ratios (ORs) and 95% confidence intervals (CIs) were used to measure the strength of association between the polymorphism rs141178472 and colorectal cancer risks. *P* < 0.05 was considered sufficient for statistical significance.

## 3. Results

### 3.1. General Characteristics of the Subjects

The distributions of selected variables between cases and controls are summarized in Table 1. Briefly, there were no significant differences in the distributions of age (*P* = 0.506), sex (*P* = 0.563), and smoking status (*P* = 0.536) between the cases and controls. However, colorectal cancer cases were significantly more likely to report a family history of cancer than the controls in their first-degree relatives (*P* = 0.023). Among 213 colorectal cancer cases, 212 (54.9%) had colon cancer and 174 (45.1%) had rectal cancer. Regarding tumor stage, 38, 173, 135, and 40 patients were classified as stages I, II, III, and IV, respectively.

### 3.2. rs141178472 Genotype Frequencies in Cases and Control

The observed genotype frequencies of rs141178472 genetic variants sites were corresponded to Hardy-Weinberg equilibrium in controls (*χ* ^2^ = 0.139, *P* = 0.709). The allelic and genotypic frequencies of the genetic variant rs141178472 were shown in Table 2. The T allele of rs141178472 genetic variant was the predominant allele in the studied subjects. As for rs141178472 C>T, significant differences were detected between the allele frequencies of CRC cases (C, 37.3%; T, 62.7%) and those of the healthy controls (C, 32.1%; T, 67.9%). In this study, we did not observe any association of relation between rs141178472 genotype and tumor stage. In addition, individuals carrying the CC genotypes for the rs141178472 were significantly associated with increased risk of CRC comparing with those carrying wild-type homozygous TT genotypes (OR (95% CI): 1.716 (1.084–2.716), *P* = 0.022). The risk of CRC was significantly higher among subjects carrying at least one C allele than among patients carrying T allele (OR (95% CI): 1.258 (1.021–1.551), *P* = 0.033).

### 3.3. Effect of the PIK3CA 3′-UTR Polymorphism rs141178472 on PIK3CA Expression

**Figure 1:**
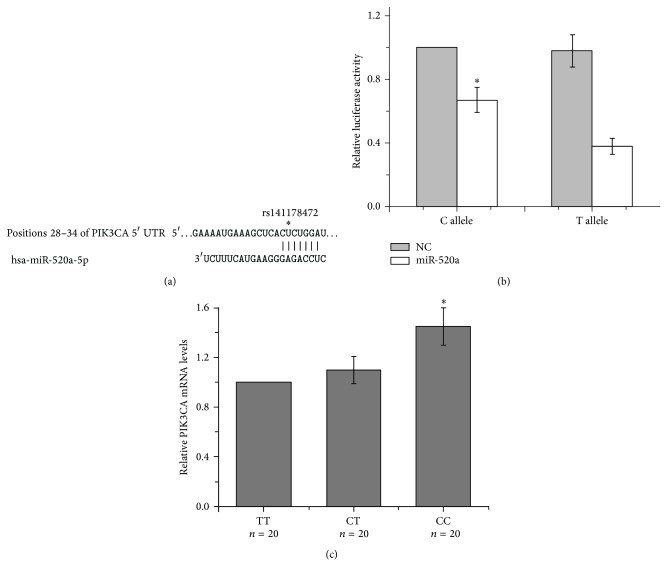
Effect of the PIK3CA 3′-UTR polymorphism rs141178472 on PIK3CA expression. (a) The sequence complementarity of hsa-miR-520a and PIK3CA 3′-UTR is shown here. (b) Luciferase activity of plasmids containing rs141178472 T or C allele in 293T cells. PIK3CA 3′-UTR luciferase reporter plasmids were cotransfected with chemically synthesized mature hsa-miR-520a or negative control (NC) in 293T cells. (c) The PIK3CA mRNA levels in PBMCs of CRC patients were detected by qRT-PCR assay. ^*^Compared  to  TT  genotype, *P* < 0.05.

The rs141178472 polymorphism located at the binding site of miR-520a in the PIK3CA 3′-UTR (Figure 1(a)). As predicted using bioinformatics analysis, rs141178472 with T allele can create a new miR-520a binding site. Thus, we hypothesized that the variant T allele might lead to a reduced expression of PIK3CA resulting from increased miRNA repression. To test this hypothesis, two luciferase reporter gene plasmids containing rs141178472 T or C allele were constructed to determine whether this SNP could affect the expression of PIK3CA (Figure 1(b)). The luciferase activity of reporter gene with rs141178472 T allele was significantly lower as compared with C allele when we cotransfected chemically synthesized mature miR-520a into 293T cell (*P* < 0.05). Furthermore, we detected the PIK3CA mRNA levels in PBMCs of CRC patients. The results of qRT-PCR assay indicated that CRC patients with CC genotype had a higher PIK3CA mRNA level than that in patients with TT genotype (Figure 1(c)).

## 4. Discussion

In the present study, we observed an association between PIK3CA 3′-UTR polymorphism rs141178472 and risk of colorectal cancer. Furthermore, we found the polymorphism rs141178472 could affect the expression of PIK3CA. Together, our findings indicate that the miR-520a/PIK3CA axis may play a role in colorectal carcinogenesis.

An increasing body of evidence indicated that a number of cancer-associated genes can be regulated by microRNAs (miRNAs). miRNAs bind to the 3′-untranslated region (3′-UTR) of mRNA through the seed region and activate the degradation of mRNA, thus repressing translation [10, 11]. As the binding between mRNA and miRNA is based on complementary base pairing, a single nucleotide polymorphism (SNP) in the 3^′-^UTR or seed region would affect the binding efficiency, which may lead to change of the expression level of their target genes [12]. For example, the miR-184 binding site SNP (rs8126 T>C) in the 3′-UTR of TNFAIP2 could modulate TNFAIP2 expression and contributes to neck squamous cell carcinoma (HNSCC) susceptibility [13]. Hikami et al. found a SNP in the 3′-UTR of SPI1 is associated with increased SPI1 mRNA level and with susceptibility to SLE [14]. Similar results were also found in ovarian cancer [15] and breast cancer [16]. In this study, we enrolled 780 research subjects of Han ethnicity in China. We found that SNP rs141178472 in PIK3CA gene was related with the risk of CRC. These results indicated that rs141178472 might be a universal CRC-related SNP in Chinese Han population. Furthermore, the expression of PIK3CA was detected in the peripheral blood of CRC patients, suggesting that mRNA expression of PIK3CA might be associated with SNP rs141178472.

PIK3CA gene encodes for the catalytic p110-alpha subunit of phosphatidylinositol 3-kinase (PI3K) alpha, which plays a role of mediator in a wide spectrum of biological processes including cell proliferation, survival, proliferation, migration, and morphology [17, 18]. PI3K is a family of enzymes capable of 3-phosphorylating in response to activation of growth factors such as vascular endothelial growth factor (VEGF), epidermal growth factor (EGF), platelet-derived growth factor (PDGF), or insulin [19]. PTEN is an important tumor suppressor gene and appears to negatively control the phosphoinositide 3-kinase signaling pathway [20]. The PI3K pathway is dysregulated in various cancers through either amplification or mutation in PIK3CA or inactivation of PTEN [21]. Activating mutations of PIK3CA are found most commonly in tumors of the stomach, colorectum, brain, breast, liver, ovaries, and lung [22, 23]. There are 2 mutation hotspots in the PIK3CA gene: codons 542 and 545 of exon 9 and codon 1047 in exon 20. The prevalence of PIK3CA mutations in patients with colorectal carcinoma may vary between 7% and 32%. Some studies demonstrated that PIK3CA mutations were associated with risk of cancers. He et al. found genotype CC in locus PIK3CA rs7646409 may increase the risk of osteosarcoma in the Chinese population [24]. Kommineni et al. reported 60.46% of head and neck squamous cell carcinoma (HNSCC) patients and 26% of controls with the following mutations 1634A>C (E545A) and 3075C>T (T1025T) in the helical and kinase domains of PIK3CA [25]. In the present study, we found a microRNA binding site polymorphism rs141178472 in the PIK3CA 3′-UTR is associated with the expression and risk of colorectal cancer. Our findings indicated that polymorphisms in untranslated regions of PIK3CA may play a role in colorectal carcinogenesis.

In summary, our findings indicated that miR-520a/PIK3CA axis may be involved in colorectal carcinogenesis, and rs141178472 polymorphism in PIK3CA 3′-UTR may contribute to colorectal cancer risk. Further larger studies and mechanistic investigation into the function of miR-520a/PIK3CA axis are warranted to advance the understanding of its role in colorectal carcinogenesis.

## Figures and Tables

**Table 1:** General characteristics of colorectal cancer cases and controls.

	Cases (*n* = 386)		Controls (*n* = 394)		*P*
Age (mean ± SD), years	60.1 ± 12.3		60.7 ± 12.9		0.506
Gender					
Male	216	56.0%	229	58.1%	0.563
Female	170	44.0%	165	41.9%	
Smoking					
Never	262	67.9%	276	70.1%	0.536
Ever	124	32.1%	118	29.9	
Family history of cancer					
No	332	86.0%	360	91.4%	0.023
Yes	54	14.0%	34	8.6%	
Tumor site					
Colon	212	54.9%			
Rectum	174	45.1%			
Tumor stages					
I	38	9.8%			
II	173	44.8%			
III	135	35.0%			
IV	40	10.4%			

**Table 2:** The association between rs141178472 and CRC risk.

SNP	Genotype	Cases *n* (%)	Controls *n* (%)	Odds ratio (95% CI)	*P* value
rs141178472	TT	156 (40.4)	180 (45.7)		
	CT	172 (44.6)	175 (44.4)	1.134 (0.840–1.531)	0.444
	CC	58 (15.0)	39 (9.9)	**1.716 (1.084–2.716)**	**0.022**
	T	484 (62.7)	535 (67.9)		**0.033**
	C	288 (37.3)	253 (32.1)	**1.258 (1.021–1.551)**	
